# Study on the Mechanism of Intestinal Absorption of Epimedins A, B and C in the Caco-2 Cell Model

**DOI:** 10.3390/molecules19010686

**Published:** 2014-01-07

**Authors:** Yan Chen, Ying Wang, Jing Zhou, Xia Gao, Ding Qu, Congyan Liu

**Affiliations:** 1 Key Layboratory of New Drug Delivery System of Chinese Meteria Medica, Jiangsu Provincial Academy of Chinese Medicine, 100 Shizi Road, Nanjing 210028, China; E-Mails: wangying9021@163.com (Y.W.); happyjingzhou@126.com (J.Z.); gaoxia0218@163.com (X.G.); quding1985@hotmail.com (D.Q.); liucongyan2007@126.com (C.L.); 2 Deparment of Pharmaceutics, School of Pharmacy, Jiangsu University, Zhenjiang 212013, China

**Keywords:** epimedin A, epimedin B, epimedin C, MK571, verapamil, dipyridamole, absorption, inhibitors, Caco-2 cell monolayer model

## Abstract

*Epimedium* spp. is commonly used in Traditional Chinese Medicine. Epimedins A, B, and C are three major bioactive flavonoids found in *Epimedium* spp. that share similar chemical structures. In this study, the intestinal absorption mechanism of these three compounds was investigated using the Caco-2 cell monolayer model in both the apical-to-basolateral (A-B) and the basolateral-to-apical (B-A) direction. The absorption permeability (P_AB_) of epimedins A, B, and C were extremely low and increased as the concentration of the epimedins increased from 5 to 20 μM, but, at 40 μM, the P_AB_ values were reduced. Meanwhile, the amount of transported compounds increased in a time-dependent manner. The P_AB_ of epimedins A and C were significantly increased and efflux ratios decreased in the presence of verapamil (an inhibitor of P-glycoprotein) and dipyridamole (an inhibitor of breast cancer resistance protein) while, in the presence of MK571 (an inhibitor of multidrug resistance proteins), the absorption of epimedins A and C did not change significantly, indicating that P-gp and BCRP might be involved in the transport of epimedins A and C. The P_AB_ of epimedin B significantly increased while its secretory permeability (P_BA_) significantly decreased in the presence of dipyridamole, indicating that BCRP might be involved in the transport of epimedin B. No obvious changes in the transport of epimedin B were observed in the presence of verapamil and MK571. In summary, our results clearly demonstrate, for the first time, that poor bioavailability of these three prenylated flavonoids is the result of poor intrinsic permeability and efflux by apical efflux transporters.

## 1. Introduction

**Figure 1:**
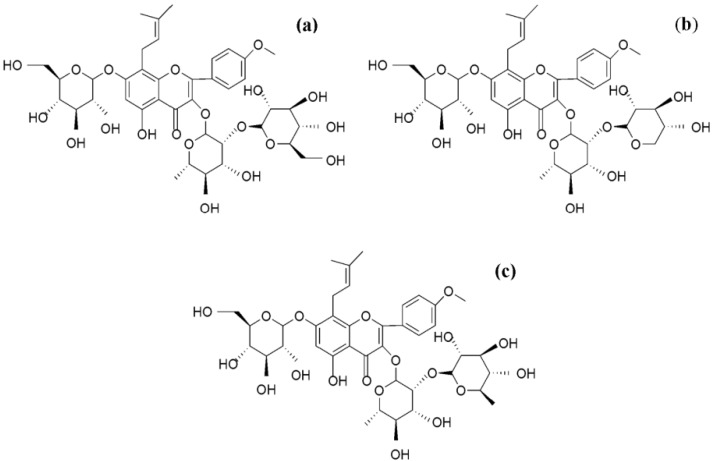
Chemical structures of (**a**) epimedin A; (**b**) epimedin B; and (**c**) epimedin C.

For over 2,000 years, *Epimedium* spp. has been widely used in China as an important medicinal herb [1]. Because of its pharmacological actions, it has been used to treat various disorders such as cardiovascular disease, osteoporosis, menopause syndrome, rheumatism, arthritis, and hypogonadism [2,3,4,5]. The main active constituents of the herb are prenylated flavonoids, among which icariin, baohuoside I, and epimedins A, B, and C are the most important (Figure 1) [6,7,8]. 

Although these flavonoids show good pharmacological actions, their bioavailability is poor. We previously investigated the mechanism of *in vitro* absorption of icariin, epimedins A, B, C, and baohuoside I by using the Caco-2 monolayer model. Our study allowed us to better define the transport mechanism of icariin and baohuoside I, although the transport mechanism of epimedins A, B, and C was not fully understood. 

The aim of present study was to further investigate the intestinal absorptive characteristics of epimedins A, B, and C by using the same Caco-2 cell model we used earlier. This model is extensively used because of its morphological and functional similarities to the human small intestinal epithelium, and it has been recognized by the FDA as a viable model that replicates human intestinal absorption [9,10,11]. Additionally, Caco-2 cells express ATP-binding cassette (*ABC*) membrane transporters that are related to flavonoid transport proteins such as P-glycoprotein (P-gp) [12,13], multidrug resistance proteins (MRPs) [14,15,16], breast cancer resistance protein (BCRP) [17,18], and organic anion transporters. In general, ABC transporters are specifically located in the apical (lumen side) or basolateral (blood/plasma side) membrane of enterocytes and facilitate the excretion back into the intestinal lumen or the uptake into the blood, respectively [19]. Therefore, the bidirectional transport (A-B and B-A) of different concentrations of epimedins A, B, and C was investigated. Moreover, by co-administering epimedins A, B, and C with different transporter inhibitors, the roles of P-gp, MRPs, and BCRP in the transport of epimedins A, B, and C were determined.

## 2. Results and Discussion

### 2.1. Cell Viability Assay

In order to determine which concentration of epimedins A, B, and C can be used in transport experiments, the *in vitro* cytotoxicity of the above compounds was analyzed by cell viability experiment. The concentration of each compound was set at 10, 20, 40, and 80 μM. An increase in the concentrations of epimedins A, B, and C was accompanied by a slight decrease in the viability of Caco-2 cells, although the difference was not statistically significant when compared to the control group. Even at the highest concentration (80 μM), the cell viability rates of Caco-2 cells treated with the three compounds reduced only by 7%, indicating that in our experimental design, epimedins A, B, and C were nontoxic to the growth of Caco-2 cells. Therefore, concentrations ranging from 5 to 40 μM were chosen for the three epimedins in the following studies.

### 2.2. Transport of Epimedins A, B, and C at Different Concentrations

**Figure 2:**
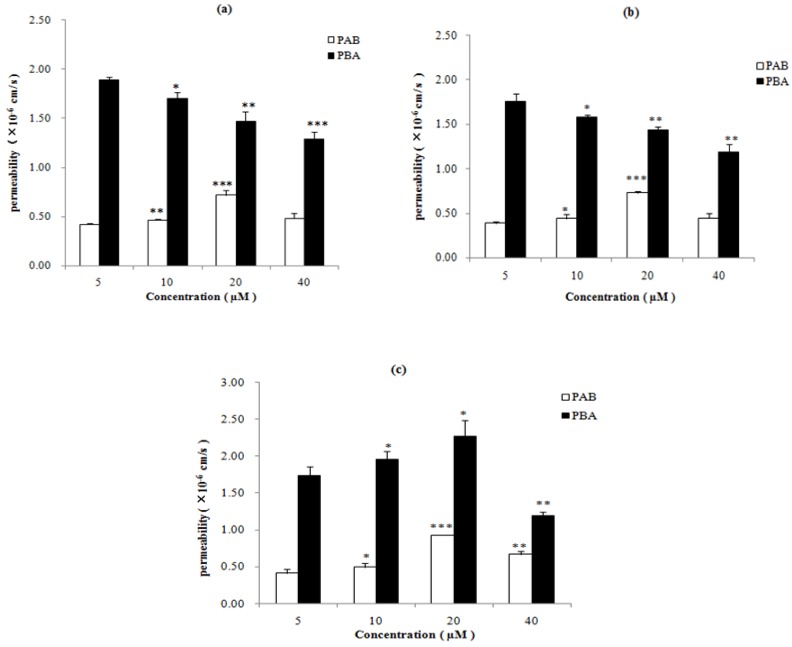
Permeability of epimedins A, B, and C at different concentrations (**a**) epimedin A, (**b**) epimedin B; and (**c**) epimedin C. In the each column figure, white column refers to the absorptive permeability (P_AB_); black column refers to secretory permeability (P_BA_). The asterisk symbol indicates a statistically significant difference between permeability at 5 μM (control) and that at a higher concentration. The number of asterisk symbol indicates the level of significance with *** *p* < 0.001, ** *p* < 0.01, * *p* < 0.05. Each data point is the average of three determinations, and the error bars represent the standard deviation of the mean.

The absorption of different concentrations (5, 10, 20, 40 μM) of epimedins A, B, and C was investigated using the Caco-2 cell model. The transport of these compounds in both the apical-to-basolateral (A-B) and the basolateral-to-apical (B-A) directions was studied, and the absorptive (P_AB_) and secretory (P_BA_) permeability of the three flavonoids were estimated (Figure 2). The P_AB_ values of epimedins A, B, and C for concentrations of 5–40 μM were 0.42–0.72 × 10^−6^ cm/s, 0.39–0.73 × 10^−6^ cm/s, and 0.42–0.72 × 10^−6^ cm/s, respectively, which were considerably lower than those of highly permeable compounds such as propranolol and testosterone [20], and were similar to those of poorly absorbed compounds such as mannitol (1.7 × 10^−6^ cm/s) and sulfasalazine (0.34 × 10^−6^) [21]. The low P_AB_ values of epimedins A, B, and C suggested that all of them presented a very poor intestinal absorption.

In contrast, the P_BA_ values of epimedins A, B, and C were significantly higher than their P_AB_ values (*p* < 0.05). The P_BA_ values of epimedins A, B, and C for concentrations of 5–40 μM were 1.29–1.89 × 10^−6^ cm/s, 1.19–1.76 × 10^−6^ cm/s, and 1.20–2.28 × 10^−6^ cm/s, respectively. As a consequence, the efflux ratios (defined as the ratio of the secretory permeability to the absorptive permeability; P_BA_/P_AB_) of epimedins A, B, and C were greater than 2 indicating that the participation of an active efflux transport of these compounds.

The transport of epimedins A, B, and C in the A-B direction was similar. As the concentration increased from 5 to 20 μM, the absorptive permeability increased, but at 40 μM, the P_AB_ value decreased, indicating that the epimedin transport is concentration-dependent, and that saturation might have been reached at 40 μM. However, transport of the three epimedins in the B-A direction was different. For epimedins A and B, P_BA_ decreased significantly (*p* < 0.05) as the concentration increased from 5 to 40 μM. For epimedin C, P_BA_ increased as the concentration increased from 5 to 20 μM, but decreased at 40 μM. This might relate to the chemical structures of the three compounds. While all of them possess identical aglycones, they are connected by different sugar moieties.

**Figure 3:**
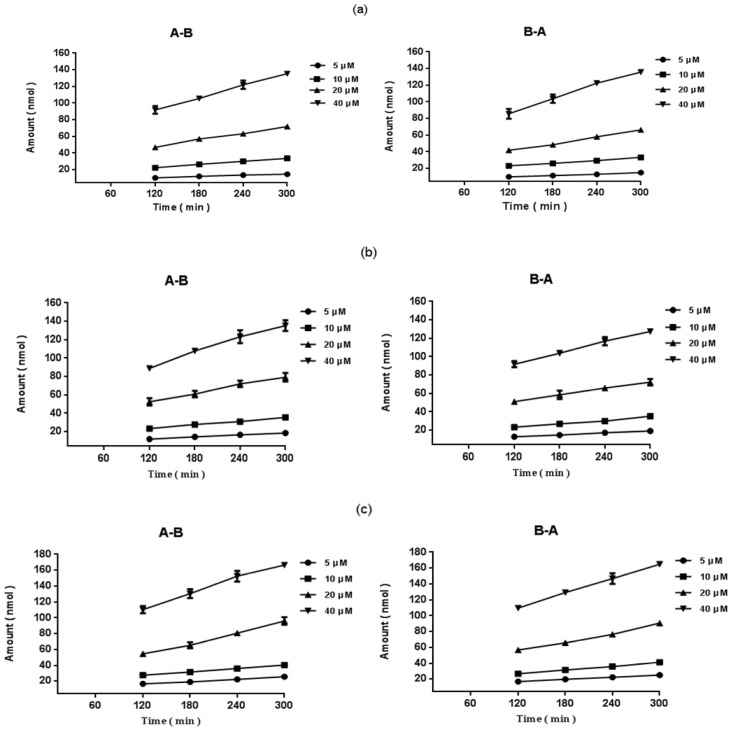
Accumulation of epimedins A, B, and C in Caco-2 cells with time. Caco-2 cell monolayers were incubated with different concentrations of epimedins A, B, and C in the apical to basolateral (A-B) and basolateral to apical (B-A) directions for 2–5 h. Each data point is the average of three determinations, and the *error bars* represent the standard deviation of the mean. (**a**) Epimedin A; (**b**) Epimedin B; and (**c**) Epimedin C.

In addition, regardless of the direction of the transport of epimedins A, B, and C, the amount of transported compound increased linearly with time (Figure 3). At the end of the transport experiments, the integrity of the monolayers was monitored using the transepithelial resistance (TEER) value, the percentage of deviation was less than 2%, and no significant changes were observed.

### 2.3. Effect of Inhibitors on the Absorption of Epimedins A, B, and C

**Figure 4:**
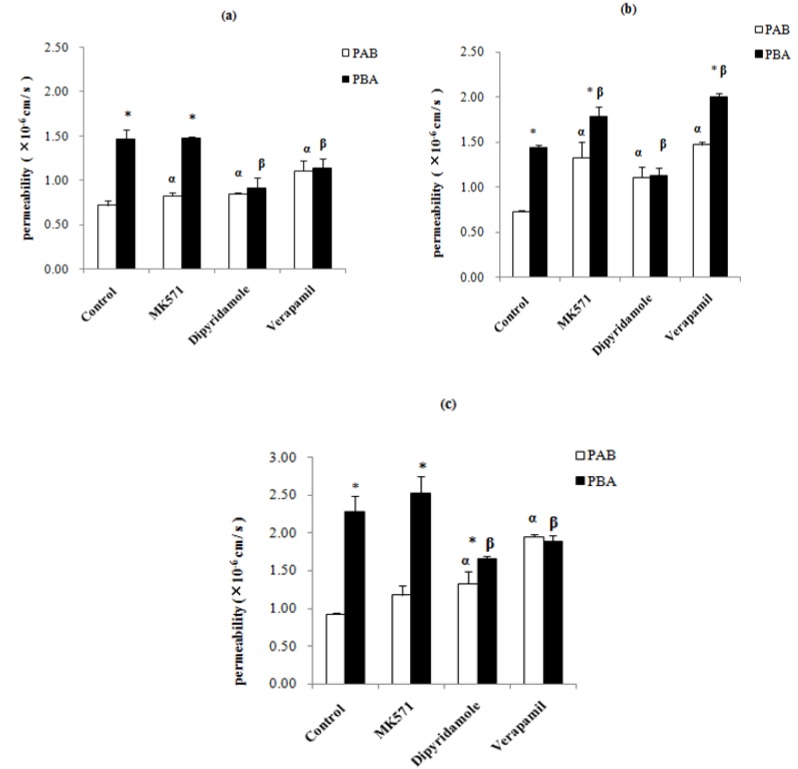
Effects of various potential inhibitors on permeabilities of epimedins A (20 μM), B (20 μM), and C (20 μM). (**a**) epimedin A; (**b**) epimedin B; and (**c**) epimedin C. In the each column figure, white column refers to the absorptive permeability (P_AB_); black column refers to secretory permeability (P_BA_). The asterisk symbol indicates that P_AB_ and P_BA_ are significantly different (*p* < 0.05) from each other. The symbol alpha indicates that the P_AB_ of epimedin A, epimedin B and epimedin C in the presence of an inhibitor is significantly different (*p* < 0.01) from that of control, whereas the symbol beta indicates the same for its P_BA_. Each data point is the average of three determinations, and the *error bars* represent the standard deviation of the mean.

Our results showed that the P_AB_ values of epimedins A, B, and C were very low. In contrast, the P_BA_ values of the epimedins were significantly higher than their P_AB_ values (*p* < 0.05), indicating that some transporters might be involved in the transport of the three compounds in B-A direction Therefore, three *ABC* transporter inhibitors, one p-glycoprotein inhibitor (verapamil), one multidrug resistance-related protein inhibitor (MK571), and one breast cancer resistance protein inhibitor (dipyridamole) were used to determine the transporters involved in the transepithelial transport of epimedins A, B, and C. When an inhibitor is highly effective against an efflux transporter, the value of the efflux ratio is expected to decrease significantly. As shown in Figure 4a, in the presence of 50-μM verapamil, the P_AB_ value of 20-μM epimedin A was 1.5-fold that of the control group, whereas its P_BA_ value was reduced by 22%, resulting a reduction in efflux ratio from 2.04 to 1.03. In the presence of 50-μM dipyridamole, the P_AB_ value of 20-μM epimedin A increased 1.2-fold, while its P_BA_ value reduced by 38%, resulting in a 47% reduction in the efflux ratio. Co-treatment with 50-μM MK571 did not alter epimedin A’s P_AB_ or the P_BA_ values, and the efflux ratio reduced only by 13%. These results suggest that the transport of epimedin A could be affected by P-gp and BCRP.

As shown in Figure 4b, epimedin B inhibition studies using verapamil and MK571 in the presence of 20-μM epimedin B showed that both P_AB_ and P_BA_ increased, with the efflux ratios being reduced by 31% and 32%, respectively. However, in presence of 50-μM dipyridamole, the absorptive permeability increased significantly, whereas the secretory permeability significantly decreased, resulting in a 50% reduction in the efflux ratio (from 1.98 to 1.02). These results indicated that BCRP might be involved in the transport of epimedin B.

The same inhibitors were used to determine the effect of each inhibitor on the transepithelial transport of 20-μM epimedin C. The results demonstrated that verapamil and dipyridamole significantly increased the absorptive permeability, but decreased the secretory permeability of epimedin C (Figure 4c). In particular, the secretory transport of epimedin C was inhibited by 17% by 50-μM verapamil and by 27% by 50-μM dipyridamole. In contrast, 50-μM verapamil induced a 109% increase in epimedin C absorptive permeability, while 50-μM dipyridamole increased it by 43%. As a result, the efflux ratio of epimedin C was reduced by approximately 2.5-fold in the presence of verapamil, and by about 1.95-fold in the presence of dipyridamole. In contrast, MK571 did not significantly influence the transport of epimedin C. These results suggest that P-gp and BCRP may affect the transport of epimedin C.

Taken together, our present study deeply investigated the intestinal absorptive characteristics of epimedins A, B, and C by using Caco-2 cell model. Although Caco-2 cell model might have some limitations as a model for predicting the intestinal absorption, such as overexpressing P-glycoprotein in relation to human enterocytes, however, up to now, Caco-2 cell model is still one of the two most popular methods for determining drug absorption mechanism and is well recognized by FDA. The special feature of this model is that both apical and basolateral sides of the intestinal epithelium are easily accessible and therefore is excellent model for studying drug excretion or efflux. At present, there are many reports on the absorption mechanism of drugs using this model. For instance, Mease *et al.* used Caco-2 cell to evaluate the selectivity of efflux transporter inhibitors such as zosuquidar, fumitremorgin C in Caco-2 cells [17], and Xia *et al.* examined the expression, localization, and functional characteristics of BCRP in Caco-2 cells [22]. Kobayashi *et al.* evaluated the absorption mechanisms of the isoflavones genistein and daidzein their microbial metabolities dihydrogenistein (DHG) and dihydrodaidzein (DHD) using the Caco-2 cell model [23].

In terms of experiments, the result demonstrated that Epimedins A, B, and C are likely to be poorly bioavailable because they have low absorptive permeability, which were similar to those of poorly absorbed compounds such as mannitol and sulfasalazine [21]. The amount of transported compound increased linearly with time indicating that the absorptive mechanisms of epimedins A, B, and C might be passive diffusion, and the same phenomenon could be found in other flavonoids [24,25]. The low PAB appears to be the result of poor intrinsic permeability, which is further limited by apical efflux transporters such as MRP2, BCRP and P-gp. The P-gp and BCRP are located at apical side in the cell membrane [26], whereas MRP has different isoforms, from which MRP2 is located at apical side in the cell membrane [27]. In the experiment, we used verapamil, dipyridamole and MK571 as inhibitor of P-gp, BCRP and MRP2, respectively, and added them at the donor side. We found that when we added the inhibitor of BCRP, the trasnsport of epimedins A, B and C from A-B direction significantly increased and the trasnsport from B-A direction significantly inhibited, while added the inhibitor of P-gp, only epimedins A and C had the same change. The significant difference between P_AB_ and P_BA_ of these three prenylated flavonoids suggested that P-gp and BCRP might be involved in the transport of epimedins A and C while BCRP might be involved in the transport of epimedin B. Therefore, poor intrinsic permeability and apical efflux of P-gp and BCRP might be the main reasons who lead to the poor bioavailability of these three prenylated flavonoids.

## 3. Experimental

### 3.1. Materials and Reagents

Cloned Caco-2 TC7 cells were kindly donated by Professor Ming Hu (University of Houston, Houston, TX, USA). Epimedin A, epimedin B and epimedin C (all purity >98%) were purchased from Yuanye Bio-Technology Co Ltd (Shanghai, China). Verapamil, MK571, Hanks’ balanced salt solution (HBSS, powder form) and testosterone (internal standard) were obtained from Sigma-Aldrich (St. Louis, MO, USA). Dipyridamole was purchased from Cayman Chemical Company (Ann Arbor, MI, USA). Fetal bovine serum was purchased from HyClone (Logan, UT, USA). Milli-Q water (Millipore, Bedford, MA, USA) was used in the study. Acetonitrile was of chromatographic grade (Merck Company Inc., Whitehouse Station, NJ, USA). All other reagents (typically analytical grade or better) were used as received.

### 3.2. Cell viability Assay

Cell viability was measured using the 3-(4, 5-dimethylthiazol-2-yl)-2,5-diphenyltetrazolium bromide (MTT) assay. The Caco-2 cells were seeded in 96-well plates at a density of 2 × 10^3^ cells/well in DMEM culture medium and cultured at 37 °C for 24 h. After that, the culture medium was replaced with fresh medium containing epimedins A, B, and C at concentrations ranging from 10 μM to 80 μM. Control groups consist of cells in medium (without epimedins A, B, and C) which are processed identically and incubated simultaneously as treated groups. And then the 96-well plates were incubated for 36 h. Subsequently, 10 μL of 5 mg/mL MTT was added to each well and the plates were incubated for another 4 h. The solutions in each well were then removed followed by dissolving the remained formazan crystals in the cells with 100 μL DMSO. The absorbance was measured at 550 nm using a microplate reader (Thermo Labsystems, Helsinki, Finland). The cell viability of each compound was calculated as the percentage of the absorbance relative to that of negative control.

### 3.3. Cell Culture

The Caco-2 TC7 cell line (at passage 30) is generally similar to the wild-type Caco-2 cell line. However, it is more stable during transportation because it is a cloned cell line [28]. The conditions for Caco-2 cell culture have been described previously [9,29,30,31]. Cells were cultured in a humidified atmosphere of 5% CO_2_ and 95% air at 37 °C. The culture medium (Dulbecco’s modified Eagle’s medium ) was supplemented with 10% (v/v) fetal bovine serum, 1% nonessential amino acids, 100 U/mL penicillin, and 100 µg/mL streptomycin. When the cell culture reached 80% confluence, it was rinsed with phosphate-buffered saline and split using trypsin. For transport experiments, the cells were seeded on 3 μm porous polycarbonate cell culture Transwell^®^ inserts from Nunc, which has a surface area of 4.2 cm^2^ at a density of 100,000 cells/cm^2^. The culture media was replaced every other day. The monolayers were ready for experiments from 19 to 21 days after seeding. Transepithelial electrical resistance (TEER) was used to monitor the integrity and tight junction of the Caco-2 cell monolayer. Only monolayers that demonstrated a TEER value above 250 Ω × cm^2^ were used in the transport experiment [32].

### 3.4. Sample Preparation

Epimedin A, epimedin B, and epimedin C were dissolved in dimethyl sulfoxide (DMSO)-ethanol (v/v = 6/4) to prepare the stock solution (8 mM) for each single compound. The above-mentioned solutions were further diluted with HBSS solution (pH = 7.4) to obtain a series of working standard solutions, and the final concentrations of epimedin A, epimedin B, and epimedin C in the transport samples were 5 µM, 10 µM, 20 µM and 40 µM, respectively. Moreover, the final concentrations of the organic solvent of different samples were controlled below 0.5% to ensure the safety to the cells.

Meanwhile, verapamil was dissolved in water to 2 mM as well as MK571 and dipyridamole were dissolved in dimethyl sulfoxide (DMSO)-ethanol (v/v = 1/1) to prepare the stock solution (10 mM) when they were used. In addition, testosterone (internal standard) was dissolved in acetonitrile with acetic acid (v/v = 94/6) to 100 µM.

### 3.5. Transport Experiments through Caco-2 Cell Culture Model

The transport experiments were performed as described previously [2,3,4,5]. Firstly, the cell monolayers were rinsed thrice with 37 °C HBSS (pH 7.4), and the monolayers were incubated with the buffer for 1 h, then the incubation medium was aspirated. Afterwards, transport from the apical side (AP) to basolateral side (BL): 2.9 mL drug solution were added to the AP side (which was regarded as donor side) and 2.5mL HBSS were added to the BL side (which was regarded as receiver side). Transport from the basolateral side to apical side: 2.9 mL drug solution were added to the BL (donor) side and 2.5 mL HBSS were added to the AP (receiver) side. When the transport inhibitors were used, they were loaded only at the donor side. In the test for each solution, four donor samples (400 μL) and four receiver samples (400 μL) were taken at time intervals of 2, 3, 4, and 5 h after incubation, followed by an immediate replacement with fresh donor solution (400 μL) to the donor side or fresh buffer (400 μL) to the receiver side. Our preliminary experiments indicated that these three compounds have low absorptive permeability, and the transport amounts of these three compounds were difficult to detect by UPLC after transport 1 h, so we took the first aliquot after 2 h in order to ensure the accuracy of the experiment.

To each transport sample (400 µL), acetonitrile (100 µL) containing 100 µM of testosterone was added as an internal standard and preservation. The resulting mixture was vortexed for 30 sec and then centrifuged at 15,000 rpm for 15min. The supernatant obtained was determined by ultra-performance liquid chromatography (UPLC) within 24 h. At the end of the transport experiment, the TEER value was remeasured to confirm the integrity of the cell monolayer [33].

### 3.6. UPLC Analysis of Transport Samples

UPLC was used to determine the concentration of the compounds in the transport samples obtained from the Caco-2 model. The conditions for UPLC analysis of epimedin A, epimedin B, and epimedin C in the presence of inhibitors (verapamil or MK571) were as follows: system, Waters Acquity^TM^ UPLC with photodiode array detector and Empower software; column, Acquity UPLC BEH C_18_, 1.7 μm, 2.1 × 50 mm (Waters, Milford, MA, USA); mobile phase A, acetonitrile; mobile phase B, water; gradient, 0 to 0.5 min, 25% A, 0.5 to 1.0 min, 25% to 60% A, 1.0 to 2.5 min, 60% to 80% A, 2.5 to 3 min, 80% to 25% A; flow rate, 0.4 mL/min; wavelength, 270 nm (epimedin A, epimedin B and epimedin C), 283nm (MK571), 278 nm (verapamil) and 245 nm (testosterone); column temperature, 30 °C; and injection volume, 5 μL. The retention times for epimedin A, epimedin B, epimedin C, MK571, verapamil and internal standard were 1.103, 1.107, 1.134, 1.820, 2.399 and 1.478 min, respectively.

While analysis the samples in the presence of another inhibitor (dipyridamole), the conditions were as follows: system and column were the same as before; mobile phase A, 90% acetonitrile, 10%water; mobile phase B, 10% acetonitrile, 90% water containing 0.015% (v/v) formic acid and 0.02% (v/v) triethylamine (pH 3.0); gradient, 0–0.3 min, 95% B, 0.3–2.9 min, 95%–65% B, 3.8 min, 0% B, 3.8–4.5 min, 0%–95% B; flow rate, 0.4 mL/min; wavelength, 270 nm (epimedin A, epimedin B and epimedin C), 285 nm (dipyridamole) and 245 nm (testosterone,); column temperature, 30 °C; and injection volume, 5 μL. The retention times for epimedin A, epimedin B, epimedin C, dipyridamole and testosterone were 2.904, 2.696, 2.630, 2.726 and 3.605 min, respectively.

In general, these methods were selective and reproducible with day to day variability less than 2%. The tested linear response ranges for all compouds were 0.3125 to 40 μM. The accuracy and precision were greater than 98%. The recoveries of three different concentrations of above mentioned compounds were around 97.25%–99.08%, and RSD were less than 5%.

### 3.7. Data Analysis

In the Caco-2 cell model, rate of transport is obtained from amount transported *versus* time curve using linear regression. The permeability of a compound is calculated using the following equation (1):

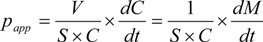
(1)
where V is the volume of the receiver (2.5 mL); S is the surface area of the cell monolayer (4.2 cm^2^) and C is the initial concentration in the donor solution (μM); hence, dC/dt is the rate of concentration change in the receiver side and dM/dt is the rate of drug transport. The rate of drug transport was calculated by linear regression analysis using Microsoft Excel software (Microsoft Corporation, Redmond, WA, USA ). All data were presented as means ± SD. Statistical comparisons were evaluated by ANOVA test using the SPSS 16 software. Results were considered significant at *p* < 0.05.

## 4. Conclusions

We investigated the intestinal absorption—by using the Caco-2 cell model—of three major bioactive flavonoids (epimedins A, B, and C) extracted from *Epimedium* spp. To our knowledge, this is the first time it has been shown that poor bioavailability of the three prenylated flavonoids is attributable to poor intrinsic permeability and efflux by apical efflux transporters such as P-gp, BCRP, and MRPs.
